# Improving Bioscience Research Reporting: The ARRIVE Guidelines for Reporting Animal Research

**DOI:** 10.1371/journal.pbio.1000412

**Published:** 2010-06-29

**Authors:** Carol Kilkenny, William J. Browne, Innes C. Cuthill, Michael Emerson, Douglas G. Altman

**Affiliations:** 1 The National Centre for the Replacement, Refinement and Reduction of Animals in Research, London, United Kingdom; 2 School of Veterinary Science, University of Bristol, Bristol, United Kingdom; 3 School of Biological Sciences, University of Bristol, Bristol, United Kingdom; 4 National Heart and Lung Institute, Imperial College London, United Kingdom; 5 Centre for Statistics in Medicine, University of Oxford, Oxford, United Kingdom

In the last decade the number of bioscience journals has increased enormously, with many filling specialised niches reflecting new disciplines and technologies. The emergence of open-access journals has revolutionised the publication process, maximising the availability of research data. Nevertheless, a wealth of evidence shows that across many areas, the reporting of biomedical research is often inadequate, leading to the view that even if the science is sound, in many cases the publications themselves are not “fit for purpose,” meaning that incomplete reporting of relevant information effectively renders many publications of limited value as instruments to inform policy or clinical and scientific practice [1]–[21]. A recent review of clinical research showed that there is considerable cumulative waste of financial resources at all stages of the research process, including as a result of publications that are unusable due to poor reporting [22]. It is unlikely that this issue is confined to clinical research [2]–[14],[16]–[20].

Failure to describe research methods and to report results appropriately therefore has potential scientific, ethical, and economic implications for the entire research process and the reputation of those involved in it. This is particularly true for animal research, one of the most controversial areas of science. The largest and most comprehensive review of published animal research undertaken to date, to our knowledge, has highlighted serious omissions in the way research using animals is reported [5]. The survey, commissioned by the National Centre for the Replacement, Refinement and Reduction of Animals in Research (NC3Rs), a UK Government-sponsored scientific organisation, found that only 59% of the 271 randomly chosen articles assessed stated the hypothesis or objective of the study, and the number and characteristics of the animals used (i.e., species/strain, sex, and age/weight). Most of the papers surveyed did not report using randomisation (87%) or blinding (86%) to reduce bias in animal selection and outcome assessment. Only 70% of the publications that used statistical methods fully described them and presented the results with a measure of precision or variability [5]. These findings are a cause for concern and are consistent with reviews of many research areas, including clinical studies, published in recent years [2]–[22].

## Good Reporting Is Essential for Peer Review and to Inform Future Research

Scrutiny by scientific peers has long been the mainstay of “quality control” for the publication process. The way that experiments are reported, in terms of the level of detail of methods and the presentation of key results, is crucial to the peer review process and, indeed, the subsequent utility and validity of the knowledge base that is used to inform future research. The onus is therefore on the research community to ensure that their research articles include all relevant information to allow in-depth critique, and to avoiding duplicating studies and performing redundant experiments. Ideally scientific publications should present sufficient information to allow a knowledgeable reader to understand what was done, why, and how, and to assess the biological relevance of the study and the reliability and validity of the findings. There should also be enough information to allow the experiment to be repeated [23]. The problem therefore is how to ensure that all relevant information is included in research publications.

## Using Reporting Guidelines Measurably Improves the Quality of Reporting

Evidence provided by reviews of published research suggests that many researchers and peer reviewers would benefit from guidance about what information should be provided in a research article. The CONSORT Statement for randomised controlled clinical trials was one of the first guidelines developed in response to this need [24],[25]. Since publication, an increasing number of leading journals have supported CONSORT as part of their instructions to authors [26],[27]. As a result, convincing evidence is emerging that CONSORT improves the quality and transparency of reports of clinical trials [28],[29].

Following CONSORT, many other guidelines have been developed—there are currently more than 90 available for reporting different types of health research, most of which have been published in the last ten years (see http://www.equator-network.org and references [30],[31]). Guidelines have also been developed to improve the reporting of other specific bioscience research areas including metabolomics and gene expression studies [32]–[37]. Several organisations support the case for improved reporting and recommend the use of reporting guidelines, including the International Committee of Medical Journal Editors, the Council of Science Editors, the Committee on Publication Ethics, and the Nuffield Council for Bioethics [38]–[41].

## Improving the Reporting of Animal Experiments—The ARRIVE Guidelines

Most bioscience journals currently provide little or no guidance on what information to report when describing animal research [42]–[50]. Our review found that 4% of the 271 journal articles assessed did not report the number of animals used anywhere in the methods or the results sections [5]. Reporting animal numbers is essential so that the biological and statistical significance of the experimental results can be assessed or the data reanalysed, and is also necessary if the experimental methods are to be repeated. Improved reporting of these and other details will maximise the availability and utility of the information gained from every animal and every experiment, preventing unnecessary animal use in the future. To address this, we led an initiative to produce guidelines for reporting animal research. The guidelines, referred to as ARRIVE (Animals in Research: Reporting *In Vivo* Experiments), have been developed using the CONSORT Statement as their foundation [24],[25].

The ARRIVE guidelines consist of a checklist of 20 items describing the minimum information that all scientific publications reporting research using animals should include, such as the number and specific characteristics of animals used (including species, strain, sex, and genetic background); details of housing and husbandry; and the experimental, statistical, and analytical methods (including details of methods used to reduce bias such as randomisation and blinding). All the items in the checklist have been included to promote high-quality, comprehensive reporting to allow an accurate critical review of what was done and what was found.

Consensus and consultation are the corner-stones of the guideline development process [51]. To maximise their utility, the ARRIVE guidelines have been prepared in consultation with scientists, statisticians, journal editors, and research funders. We convened an expert working group, comprising researchers and statisticians from a range of disciplines, and journal editors from *Nature Cell Biology*, *Science*, *Laboratory Animals*, and the *British Journal of Pharmacology* (see Acknowledgments). At a one-day meeting in June 2009, the working group agreed the scope and broad content of a draft set of guidelines that were then used as the basis for a wider consultation with the scientific community, involving researchers, and grant holders and representatives of the major bioscience funding bodies including the Medical Research Council, Wellcome Trust, Biotechnology and Biological Sciences Research Council, and The Royal Society (see Table 1). Feedback on the content and wording of the items was incorporated into the final version of the checklist. Further feedback on the content utility of the guidelines is encouraged and sought.

**Table 1:** Funding bodies consulted.

Name of Bioscience Research Funding Body
Medical Research Council
Biotechnology and Biological Sciences Research Council
Wellcome Trust
The Royal Society
Association of Medical Research Charities
British Heart Foundation
Parkinson's Disease Society

The ARRIVE guidelines (see Table 2) can be applied to any area of bioscience research using laboratory animals, and the inherent principles apply not only to reporting comparative experiments but also to other study designs. Laboratory animal refers to any species of animal undergoing an experimental procedure in a research laboratory or formal test setting. The guidelines are not intended to be mandatory or absolutely prescriptive, nor to standardise or formalise the structure of reporting. Rather they provide a checklist that can be used to guide authors preparing manuscripts for publication, and by those involved in peer review for quality assurance, to ensure completeness and transparency.

**Table 2:** Animal Research: Reporting *In Vivo* experiments: The ARRIVE guidelines.

	ITEM	RECOMMENDATION
**TITLE**	1	Provide as accurate and concise a description of the content of the article as possible.
**ABSTRACT**	2	Provide an accurate summary of the background, research objectives (including details of the species or strain of animal used), key methods, principal findings, and conclusions of the study.
**INTRODUCTION**		
**Background**	3	a. Include sufficient scientific background (including relevant references to previous work) to understand the motivation and context for the study, and explain the experimental approach and rationale.b. Explain how and why the animal species and model being used can address the scientific objectives and, where appropriate, the study's relevance to human biology.
**Objectives**	4	Clearly describe the primary and any secondary objectives of the study, or specific hypotheses being tested.
**METHODS**		
**Ethical statement**	5	Indicate the nature of the ethical review permissions, relevant licences (e.g. Animal [Scientific Procedures] Act 1986), and national or institutional guidelines for the care and use of animals, that cover the research.
**Study design**	6	For each experiment, give brief details of the study design, including:a. The number of experimental and control groups.b. Any steps taken to minimise the effects of subjective bias when allocating animals to treatment (e.g., randomisation procedure) and when assessing results (e.g., if done, describe who was blinded and when).c. The experimental unit (e.g. a single animal, group, or cage of animals).A time-line diagram or flow chart can be useful to illustrate how complex study designs were carried out.
**Experimental procedures**	7	For each experiment and each experimental group, including controls, provide precise details of all procedures carried out. For example:a. How (e.g., drug formulation and dose, site and route of administration, anaesthesia and analgesia used [including monitoring], surgical procedure, method of euthanasia). Provide details of any specialist equipment used, including supplier(s).b. When (e.g., time of day).c. Where (e.g., home cage, laboratory, water maze).d. Why (e.g., rationale for choice of specific anaesthetic, route of administration, drug dose used).
**Experimental animals**	8	a. Provide details of the animals used, including species, strain, sex, developmental stage (e.g., mean or median age plus age range), and weight (e.g., mean or median weight plus weight range).b. Provide further relevant information such as the source of animals, international strain nomenclature, genetic modification status (e.g. knock-out or transgenic), genotype, health/immune status, drug- or test-naïve, previous procedures, etc.
**Housing and husbandry**	9	Provide details of:a. Housing (e.g., type of facility, e.g., specific pathogen free (SPF); type of cage or housing; bedding material; number of cage companions; tank shape and material etc. for fish).b. Husbandry conditions (e.g., breeding programme, light/dark cycle, temperature, quality of water etc. for fish, type of food, access to food and water, environmental enrichment).c. Welfare-related assessments and interventions that were carried out before, during, or after the experiment.
**Sample size**	10	a. Specify the total number of animals used in each experiment and the number of animals in each experimental group.b. Explain how the number of animals was decided. Provide details of any sample size calculation used.c. Indicate the number of independent replications of each experiment, if relevant.
**Allocating animals to experimental groups**	11	a. Give full details of how animals were allocated to experimental groups, including randomisation or matching if done.b. Describe the order in which the animals in the different experimental groups were treated and assessed.
**Experimental outcomes**	12	Clearly define the primary and secondary experimental outcomes assessed (e.g., cell death, molecular markers, behavioural changes).
**Statistical methods**	13	a. Provide details of the statistical methods used for each analysis.b. Specify the unit of analysis for each dataset (e.g. single animal, group of animals, single neuron).c. Describe any methods used to assess whether the data met the assumptions of the statistical approach.
**RESULTS**		
**Baseline data**	14	For each experimental group, report relevant characteristics and health status of animals (e.g., weight, microbiological status, and drug- or test-naïve) before treatment or testing (this information can often be tabulated).
**Numbers analysed**	15	a. Report the number of animals in each group included in each analysis. Report absolute numbers (e.g. 10/20, not 50%^a^).b. If any animals or data were not included in the analysis, explain why.
**Outcomes and estimation**	16	Report the results for each analysis carried out, with a measure of precision (e.g., standard error or confidence interval).
**Adverse events**	17	a. Give details of all important adverse events in each experimental group.b. Describe any modifications to the experimental protocols made to reduce adverse events.
**DISCUSSION**		
**Interpretation/scientific implications**	18	a. Interpret the results, taking into account the study objectives and hypotheses, current theory, and other relevant studies in the literature.b. Comment on the study limitations including any potential sources of bias, any limitations of the animal model, and the imprecision associated with the results^a^.c. Describe any implications of your experimental methods or findings for the replacement, refinement, or reduction (the 3Rs) of the use of animals in research.
**Generalisability/translation**	19	Comment on whether, and how, the findings of this study are likely to translate to other species or systems, including any relevance to human biology.
**Funding**	20	List all funding sources (including grant number) and the role of the funder(s) in the study.

a Schulz, et al. (2010) [24].

## Improved Reporting Will Maximise the Output of Published Research

These guidelines were developed to maximise the output from research using animals by optimising the information that is provided in publications on the design, conduct, and analysis of the experiments. The need for such guidelines is further illustrated by the systematic reviews of animal research that have been carried out to assess the efficacy of various drugs and interventions in animal models [8],[9],[13],[52]–[55]. Well-designed and -reported animal studies are the essential building blocks from which such a systematic review is constructed. The reviews have found that, in many cases, reporting omissions, in addition to the limitations of the animal models used in the individual studies assessed in the review, are a barrier to reaching any useful conclusion about the efficacy of the drugs and interventions being compared [2],[3].

Driving improvements in reporting research using animals will require the collective efforts of authors, journal editors, peer reviewers, and funding bodies. There is no single simple or rapid solution, but the ARRIVE guidelines provide a practical resource to aid these improvements. The guidelines will be published in several leading bioscience research journals simultaneously [56]–[60], and publishers have already endorsed the guidelines by including them in their journal Instructions to Authors subsequent to publication. The NC3Rs will continue to work with journal editors to extend the range of journals adopting the guidelines, and with the scientific community to disseminate the guidelines as widely as possible (http://www.nc3rs.org.uk/ARRIVE).

## Abbreviations

ARRIVE: Animals in Research: Reporting *In Vivo* Experiments
NC3Rs: National Centre for the Replacement, Refinement and Reduction of Animals in Research
